# Modeling the dynamics of EMT reveals genes associated with pan-cancer intermediate states and plasticity

**DOI:** 10.1038/s41540-025-00512-2

**Published:** 2025-04-10

**Authors:** MeiLu McDermott, Riddhee Mehta, Evanthia T. Roussos Torres, Adam L. MacLean

**Affiliations:** 1 Department of Quantitative and Computational Biology, Dornsife College of Letters, Arts and Sciences, University of Southern California, Los Angeles, CA USA; 2 Department of Medicine, Division of Medical Oncology, Keck School of Medicine, Norris Comprehensive Cancer Center, University of Southern California, Los Angeles, CA USA

**Keywords:** Bayesian inference, Cellular noise, Dynamical systems, Genetic interaction, Population dynamics

## Abstract

Epithelial–mesenchymal transition (EMT) is a cell state transition co-opted by cancer that drives metastasis via stable intermediate states. Here we study EMT dynamics to identify marker genes of highly metastatic intermediate cells via mathematical modeling with single-cell RNA sequencing (scRNA-seq) data. Across multiple tumor types and stimuli, we identified genes consistently upregulated in EMT intermediate states, many previously unrecognized as EMT markers. Bayesian parameter inference of a simple EMT mathematical model revealed tumor-specific transition rates, providing a framework to quantify EMT progression. Consensus analysis of differential expression, RNA velocity, and model-derived dynamics highlighted *SFN* and *NRG1* as key regulators of intermediate EMT. Independent validation confirmed *SFN* as an intermediate state marker. Our approach integrates modeling and inference to identify genes associated with EMT dynamics, offering biomarkers and therapeutic targets to modulate tumor-promoting cell state transitions driven by EMT.

## Introduction

Cell state transitions are phenotypic changes in the state of a cell, primarily driven by transcriptional programs. Such phenotypic transitions underlie development, regeneration, and cancer. Our ability to interrogate cell state transitions and their consequences has dramatically increased with advances in single-cell genomics^1^. We can dissect the timing of key events as cells change state^2^ and identify transient or intermediate states^3^. Efforts to produce a comprehensive catalog of cell states are underway^4^, yet large gaps in our understanding remain: both regarding cell states and even more regarding the transitions they undertake. We do not have satisfactory explanations of what are the initiating factors of a cell state transition, nor what is the relationship between the dynamics of cell phenotypic change and the transcriptional dynamics acting within the cell.

The epithelial-to-mesenchymal transition (EMT), during which epithelial cells become mesenchymal or mesenchymal-like^5^, is an exemplary cell state transition. EMT is necessary during development and wound healing and is co-opted by cancer, where it is a crucial component of metastasis. Understanding EMT is thus imperative to slowing or preventing metastasis, the leading cause of death from cancer^6^. Classical conceptions of EMT characterize a binary process, with cells being either completely epithelial or mesenchymal^5^. However, experimental and theoretical studies have demonstrated the existence of EMT intermediate states^7–12^. Pan-cancer studies of intermediate EMT states have revealed insight into transcriptomic signatures underlying EMT transformation^13^. The intermediate state displays partial EMT phenotypes, with characteristics of both epithelial and mesenchymal states, and may also be called partial EMT, hybrid EMT, or an E/M state^14^. EMT intermediate states are closely tied with the concept of epithelial–mesenchymal plasticity (EMP): dynamic, bidirectional transitions through multiple EMT states.

EMT intermediate states are found in both non-malignant EMT and cancer^14,15^. The relevance of targeting these states is compelling: EMT intermediate states have been associated with circulating tumor cells^16–18^ and metastasis^19^, perhaps even more potently than mesenchymal cells alone^20,21^. We focus here on stable EMT intermediate states: biologically, this refers to cells in a state that can be isolated and persist under sufficient conditions; mathematically, stability is defined via the Lyapunov exponents of a dynamical system^22^. EMT intermediate states have been described as “metastable” in the literature, which in this case refers to stable cell states with small basins of attraction. EMT intermediate state cells may be hard to observe in part due to their rarity (small population sizes or small basins of attraction) or their location (existing at the margins rather than throughout a tissue^23^), although they are not necessarily a minority of cells in a sample.

Mathematical models of EMT have predicted and identified intermediate states, using transcriptional networks that can successfully capture both the steady states of the system and its dynamic properties^9,24–28^. These transcription models of EMT, typically regulated by transforming growth factor-beta (TGF-*β*), primarily focus on a core network with transcription factors ZEB, SNAIL, and OVOL, and micro-RNAs miR200 and miR34. Although greater attention has been paid to the transcriptional dynamics, there has also been mathematical modeling of the cell population dynamics during EMT, as reviewed in ref. ^29^.

Integrating single-cell genomics with mathematical models offers means to infer dynamic properties from high-dimensional systems^30,31^. EMT, with its relatively straightforward trajectory (non-branching, non-cyclical), lends itself well to analysis via trajectory inference (pseudotime)^32^, albeit not taking into account the spatial components of the cell fate decisions which can be decidedly more complex^33^. Trajectory inference coupled with mathematical modeling has led to insight into the initiation and timing of EMT^34^. Despite limitations in inferring Markovian cell dynamics from single-cell data^35^, experimental methodologies such as metabolic labeling^36^ or lineage tracing^21^ can overcome these challenges. Here, we take an alternative approach to inferring the population dynamics model directly from data^35,37^, and (in keeping with the observation that cell state transition dynamics are non-Markovian^38^) we propose a population model of EMT cell state transitions a priori. We subsequently learn rates of cell state transition for each individual sample via Bayesian parameter inference of the cell dynamics over pseudotime.

Here we use single-cell RNA sequencing (scRNA-seq) data to fit mathematical models of EMT population dynamics across various tumor types and stimuli. Parameter inference across these different conditions reveals shared and distinct properties of the routes of EMT. We identify shared genes associated with EMT intermediate states across tumor types via differential expression and differential RNA velocity analyses. By comparing intermediate state genes with inferred EMT parameters, we identify genes associated with EMT dynamics—that is, genes that speed up or slow down EMT. We confirm top predictions by an independent analysis of EMT in a new cell type, demonstrating how these methods offer novel means to identify biomarkers or potential targets during cell state transitions.

## Results

### Single-cell analysis of EMT across cancer types & stimuli identifies a spectrum of EMT states

**Fig. 1:**
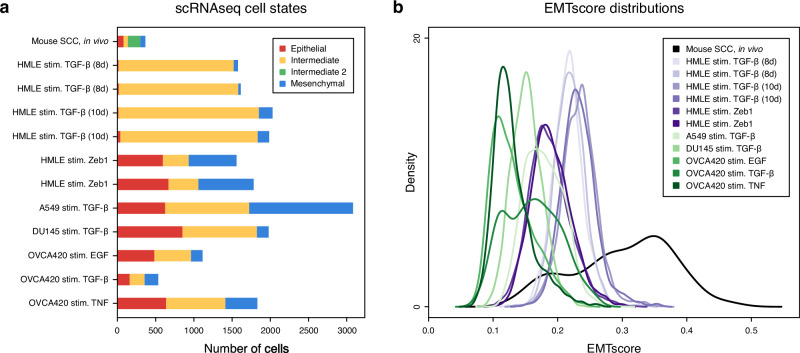
Quantification and distribution of EMT states across scRNA-seq cell lines and stimuli. **a** Cell count of EMT states per cancer sample, including intermediate states. Cell states were identified via clustering and gene expression. **b** Kernel density estimates of the EMTscore distributions for each scRNA-seq sample. Single-cell EMT scores were assigned via Hallmark EMT genes from MSigDB.

To characterize trajectories across a spectrum of EMT, we studied twelve scRNA-seq datasets across five cancer types. Cells were processed and clustered to identify cell states. We found evidence for three cell states in each of the in vitro cell populations and four states in the in vivo mouse skin squamous cell carcinoma (SCC) sample (Supplementary Figs. 1 and 2). Silhouette scores broadly support the selected clustering resolutions, balancing cluster quality and number of states (Supplementary Fig. 3). Clusters were labeled based on EMT markers from the literature, including Hallmark EMT genes from the Molecular Signatures Database (MSigDB)^39^ and epithelial cell genes from PanglaoDB^40^. Distinct clusters representing epithelial and mesenchymal cell types were identified in each dataset, although the relative sizes of these clusters varied widely (Fig. 1a). In all datasets, at least one cluster expressing combinations of epithelial and mesenchymal marker genes was identified as an intermediate state. Certain samples from ref. ^41^ that did not exhibit a clear EMT were excluded from further analyses (Supplementary Figs. 4 and 5). This is in agreement with ref. ^41^, who also found that certain conditions did not permit a full EMT within the experimental timeframe.

EMT scores were assigned to single cells across all datasets (Fig. 1b). Single cells were each assigned an EMTscore via UCell^42^ using MSigDB Hallmark EMT genes. Each sample exhibited a range of EMTscore, reproducible by replicate and varying considerably by cell type and stimulus. Notably, not only the variance but also the start and end points vary by cell type, highlighting differences not only in EMT but also in the “epithelialness” of different cell types. Samples excluded from analysis due to lack of/incomplete EMT, as identified by marker gene expression, exhibited little to no variation in EMTscore (Supplementary Fig. 5), confirming the lack of cell state transition under the tested conditions. The in vivo EMT in mouse SCC exhibited the largest range of EMTscore by a wide margin, highlighting the increase in heterogeneity among single cells during a spontaneous, unstimulated, environment-dependent EMT. Since an additional intermediate state was identified in this dataset, in line with previous work^43,44^, the data suggest that both the number of attractor states and the size of their basins of attraction are larger for cells in their natural environment than cell line-derived models stimulated in vitro.

### Shared marker genes of intermediate EMT states are associated with extracellular function

**Fig. 2:**
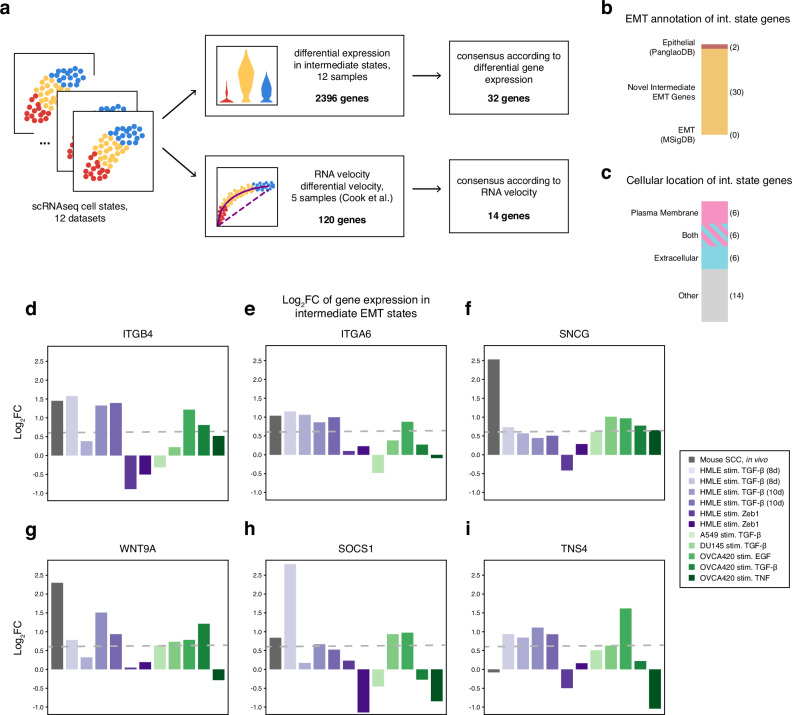
Identification of pan-cancer intermediate EMT state genes. **a** Data analysis pipeline: pan-cancer intermediate EMT marker genes were identified using differential expression and compared against genes differentially regulated via RNA velocity. **b** Annotation of predicted intermediate EMT genes by canonical epithelial/mesenchymal gene sets. **c** Annotation of predicted intermediate EMT genes by cellular location. **d**–**i** Comparison of expression of predicted intermediate EMT genes by log_2_FC (in the intermediate state) across samples. The dashed line represents 1.5-fold change (log_2_FC >0.58).

EMT can proceed along many paths^11^, and both cell/treatment-specific and consensus EMT pathways are important to study in different contexts. Here, we focus on the shared properties of EMT cell state transitions. To study intermediate state gene expression across an EMT spectrum, we performed differential gene expression and differential RNA velocity analysis across intermediate states in different cell populations (Fig. 2a). We identified differentially expressed genes for intermediate states in each sample (2396 genes total) and examined shared intermediate state-specific genes, defined as those upregulated in an intermediate state relative to epithelial/mesenchymal states. Using a log_2_ fold change (log_2_FC) threshold of +0.58 (1.5-fold change) in at least five samples, we identified 32 genes shared among EMT intermediate states (Supplementary Fig. 6 and Supplementary Data 1). No single gene is universally upregulated across intermediate EMT states; notably, the same holds for epithelial and mesenchymal states across all datasets. This observed heterogeneity is consistent with previous findings that canonical EMT genes^45^ and empirically derived EMT gene sets^46^ exhibit substantial variability, underscoring the complex & context-dependent nature of EMT.

Among the 32 genes shared across EMT intermediate states, most were absent from canonical EMT or epithelial gene sets (Fig. 2b). Two predicted intermediate state genes, *ITGB4* and *SFN*, are annotated as epithelial genes in PanglaoDB^40^, although the literature on these genes is complicated: integrin *β*4 (*ITGB4*) (Fig. 2d) was initially identified in epithelial cells and tumors^47^ but has also been linked to promoting EMT in hepatocellular and pancreatic carcinoma^48,49^. *ITGB4* pairs with another intermediate state gene, integrin *α*6 (*ITGA6*) (Fig. 2e), to form the *α*6*β*4 complex, which is implicated in promoting EMT characteristics in hepatocellular carcinoma cells^50^. Stratifin (*SFN*) (Supplementary Fig. 6) is annotated as epithelial (named for its role in the stratification of epithelial cells^51^) but is also linked to cell migration and EMT markers in cervical and hepatocellular carcinoma^52–54^. The apparent contradictory roles of both *ITGB4* and *SFN* as marking for both epithelial and mesenchymal states can be reconciled if these genes are in fact markers of an intermediate EMT state, as predicted by our analysis.

A majority of predicted intermediate EMT marker genes encode proteins localized in the extracellular space, on the plasma membrane or as secreted signaling factors (Fig. 2c). Gamma-synuclein (*SNCG*), upregulated across multiple cell lines (Fig. 2f), is found in the extracellular exosome. It plays a role in suppressing mesenchymal markers including *CDH2* (N-cadherin) and *VIM*^55^ while promoting cancer cell migration^56–58^. Other notable upregulated genes include *WNT9A*, *IL4R*, and *IL6R*. Wnt-9a (*WNT9A*) (Fig. 2g) is a secreted protein in the canonical Wnt/*β*-catenin signaling pathway that is implicated in partial EMT by mediating cell adhesion^59^. *IL4R* and *IL6R* (Supplementary Fig. 6) are interleukin cell surface receptors, with their cytokines IL4, IL13, and IL6 associated with EMT promotion^60–62^. Interestingly, *SOCS1* (suppressor of cytokine signaling 1) (Fig. 2h) is a negative regulator of IL6 yet conversely has been found to promote EMT^63^, highlighting the bidirectional signaling at play during the establishment of intermediate EMT states.

Tensin 4 (*TNS4*) (Fig. 2i) is involved in focal adhesion & integrin interaction and promotes EMT and cell motility^64,65^. Tubulointerstitial nephritis antigen-like 1 (*TINAGL1*) (Supplementary Fig. 6) encodes another secreted protein that binds directly to certain integrins, and it is found to both promote and inhibit metastasis in different cancers in vivo^66,67^. Both *TNS4* and *TINAGL1* interact with epidermal growth factor receptor *EGFR*, yet their effects are contradictory: *TNS4* reduces *EGFR* degradation^68^, while *TINAGL1* binds directly to *EGFR* and suppresses *EGFR* signaling^66^. These opposing interactions may again reflect the dynamic balance necessary to sustain the intermediate EMT state.

Overall, many genes associated with the intermediate EMT state exhibit conflicting roles in the literature, including *ITGB4*, *SFN*, *IL4R* and *IL6R* with *SOCS1*, and *TINAGL1* with *TNS4*. These genes can contribute both to the promotion and inhibition of EMT as well as the balance between epithelial and mesenchymal states. This duality underscores the dynamic nature of EMT and the importance of intermediate states. Gene set enrichment analysis (GSEA) of intermediate state genes identified enriched pathways (Supplementary Data 2), though most represented general cellular processes or were supported by only 2–3 genes. GSEA also revealed enrichment of transcription factor binding sites, particularly for AP-1, suggesting a regulatory role during transitions through EMT intermediate states.

The tumor microenvironment (TME) likely influences EMT-driven cell state transitions, as indicated by greater variability in intermediate states and EMT scores in vivo compared to in vitro (Fig. 1). We identified genes marking EMT intermediate states across all datasets (TME + non-TME) and compared them to those shared only among in vitro datasets (non-TME). Including in vivo data revealed 32 differentially expressed genes in intermediate states (Fig. 2), whereas excluding it reduced this number to 22 (Supplementary Fig. 7), hinting at the complexity the TME introduces. However, gene ontology and pathway enrichment analysis via PantherDB did not find any significant terms that distinguished TME from non-TME intermediate EMT genes, suggesting subtle regulatory influences.

### Differential regulation via RNA velocity reveals EM plasticity genes in EMT intermediate states

**Fig. 3:**
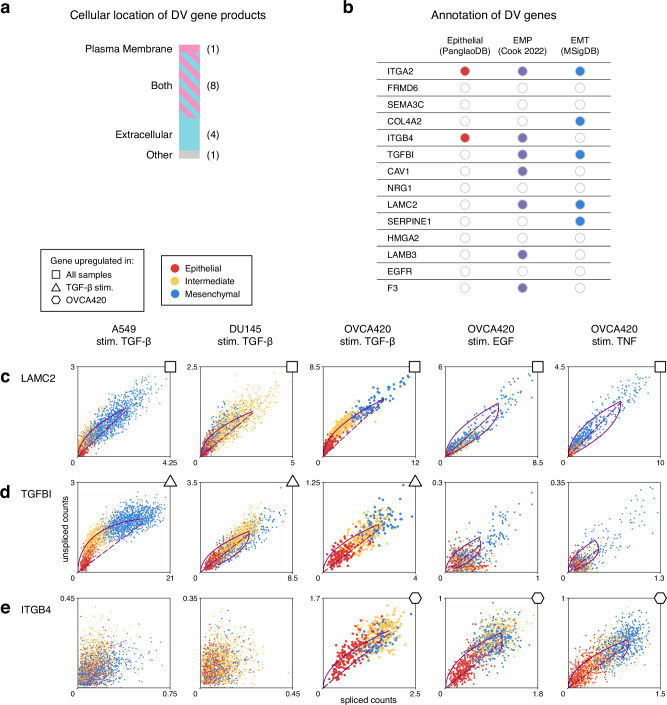
Differentially regulated intermediate EMT state genes identified via RNA velocity. **a** Annotation of cellular locations for genes differentially regulated in the intermediate state, identified through differential velocity (DV) analysis. **b** Annotation of the EMT properties of DV genes by comparison with three EMT marker gene sources. EMP: epithelial–mesenchymal plasticity. **c** Examples of DV genes upregulated in intermediate EMT states across different conditions. Solid line represents the dynamical model fit; dashed line represents the inferred steady state. *LAMC2* is upregulated across samples from different cell lines & stimuli. **d** *ITGB4* is upregulated only with a specific stimulus: TGF-*β*. **e** *ITGB4* is upregulated only in a specific tumor type: ovarian cells OVCA420.

To investigate dynamically regulated genes during EMT, we performed differential RNA velocity across EMT cell states^69,70^. Fourteen genes had differential velocity (DV) in the intermediate state in at least three of the five^41^ samples, which includes cells from lung, prostate, and ovarian tumors (Supplementary Fig. 8). Of the 14 DV genes, all but one encode proteins located extracellularly or in the plasma membrane (Fig. 3a). Several of these genes are involved in focal adhesion, including integrins *ITGA2* and *ITGB4*, laminins *LAMC2* and *LAMB3*, collagen *COL4A2*, and plasma membrane caveolae component *CAV1*. Eleven of the DV genes have annotated signal peptide sequences, underscoring their designation as secretory/membrane proteins^71,72^.

DV genes showed greater overlap with canonical EMT gene sets than the intermediate state marker genes we identified (Fig. 3b). This is expected, as genes actively upregulated in EMT intermediate states are more likely to overlap with mesenchymal markers. Epithelial–mesenchymal plasticity (EMP), i.e., bidirectional cell state transitions between epithelial and mesenchymal phenotypes^46^, is also characteristic of the DV genes identified. This overlap supports EMP conceptually: capricious cells require dynamic changes in gene expression to change state.

Comparison of DV genes across samples revealed a variety of responses: some genes were shared across different cell types and conditions, while others were specific to certain conditions. Genes upregulated regardless of cell type or stimulus included *LAMC2* (Fig. 3c), *FRMD6*, and *SERPINE1* (Supplementary Fig. 8). In contrast, and perhaps unsurprisingly, TGF-*β*-induced protein *TGFBI* (Fig. 3d) was upregulated in various cell types only when stimulated by TGF-*β*. A similar pattern was observed for *COL4A2* (Supplementary Fig. 8). Genes upregulated by multiple stimuli in one cell type, human ovarian OVCA420 cells, included *ITGB4* (Fig. 3e), *CAV1*, *HMGA2*, *F3*, and *LAMB3* (Supplementary Fig. 8). Overall, RNA velocity analysis elucidates gene regulation during EMT. Most differentially regulated genes are specific to a stimulus or cell line; fewer are conserved across conditions. There is substantial overlap between actively regulated genes during EMT and those linked to EMP, highlighting the role of dynamic transitions between cell states during EMT.

### Mathematical modeling & parameter inference quantifies EMT population dynamics

**Fig. 4:**
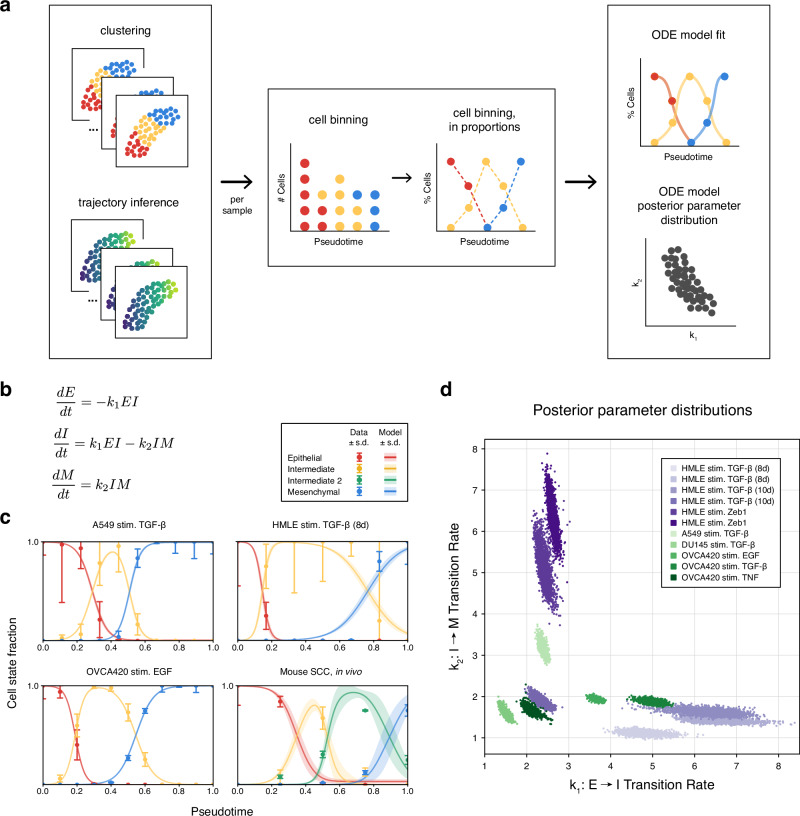
Mathematical model of EMT dynamics fitted to scRNA-seq data. **a** Workflow to infer dynamic EMT transition rates from scRNA-seq data. For each sample, clustering and trajectory inference information was processed to quantify cell states over pseudotime. A mathematical model was then fit to each sample to infer parameter posterior distributions. **b** Mathematical model representing transitions from epithelial (*E*) to intermediate (*I*) to mesenchymal (*M*) state cells. *k*_1_ is the transition rate *E* → *I*; *k*_2_ is the transition rate *I* → *M*. Additional intermediate states can be seamlessly added (Supplementary Fig. 9A). **c** Model fits the following parameter inference: data vs. trajectory simulations, with simulation parameters sampled from the posterior of each model. **d** Posterior parameter distributions of the model for each fitted sample.

Gene expression is not static: life arises from dynamics. To study the dynamics of EMT in more depth, we developed a mathematical model describing cell state transitions during EMT (Fig. 4a). The model is characterized by rate parameters for transitions between epithelial (*E*), intermediate (*I*), and mesenchymal (*M*) states, such as *E* → *I* at rate *k*_1_ (Fig. 4b and Supplementary Fig. 9A). These rate parameters were fit to scRNA-seq data, characterizing cell state transitions during EMT across pseudotime. Multiple pseudotime trajectories were calculated for each sample, rooted by different epithelial cells, to estimate the mean & variance in pseudotime based on root node selection. Cell state proportions across pseudotime, representing cell population dynamics during EMT, were fitted to the model.

EMT dynamics for each dataset were fit using Bayesian parameter inference (Fig. 4c, Supplementary Fig. 9, and Supplementary Table 2). Differences in EMT dynamics were observed across different datasets, both by cell type and by stimulus. For instance, the intermediate state persisted longer in HMLE cells compared to A549 or OVCA420 cells. Analysis of the parameter posterior distributions for each fitted EMT trajectory revealed similarities and differences in EMT dynamics (Fig. 4d). Dividing the posterior space into three approximate regions: *k*_1_ ≈ *k*_2_ (similar transition rates across EMT); *k*_1_ > *k*_2_ (faster transition rates for *E* → *I* than *I* → *M*); and *k*_1_ < *k*_2_ (faster transition rates for *I* → *M* than *E* → *I*) highlights how both cell type and stimulus can strongly impact EMT dynamics. For example, OVCA420 cells exhibited *k*_1_ > *k*_2_ dynamics regardless of stimulus, where *k*_1_ > *k*_2_ implies a larger/more stable intermediate state. In contrast, HMLE cells exhibited *k*_1_ > *k*_2_ dynamics for TGF-*β* stimulation but *k*_1_ < *k*_2_ for ZEB1 stimulation, indicating that the persistence/stability of the HMLE intermediate state depends on the stimulating factor.

An inverse proportion relationship is evident across cell types/stimuli and within a sample; this concordance is notable since more generally different types of parameter covariation can exist^30^. This analysis highlights how EMT intermediate persistence and stability depend on the intrinsic properties of the EMT experiment, with different carcinomas exhibiting greater or lesser sensitivity to EMT-inducing factors and thus affecting EMT progression.

For samples from ref. ^41^ with biological timepoints, we compared inferred pseudotime with experimental time (Supplementary Fig. 10). Pseudotime reconstructs latent dynamics by inferring a trajectory that does not necessarily align with discrete biological sampling; for instance, ref. ^41^ measured cell states at five timepoints, whereas we infer trajectories using 15 pseudotime points. In most samples, the absence of clear state transitions over experimental time precluded model fitting, underscoring the utility of pseudotime in capturing cell state dynamics. However, in two cases (A549 and OCA420 stimulated with TGF-*β*), cell state transitions occurred directly over experimental time, allowing us to fit a mathematical model to these trajectories. The lower temporal resolution of experimental sampling (two timepoints capturing the intermediate state) compared to pseudotime (four to five points) limits the precision of the intermediate state dynamics, highlighting the strength of pseudotime analysis in revealing the cell state transition dynamics that may not be observed by sparse experimental sampling.

### Consensus analysis predicts that *SFN* and *NRG1* influence intermediate cell state dynamics during EMT

**Fig. 5:**
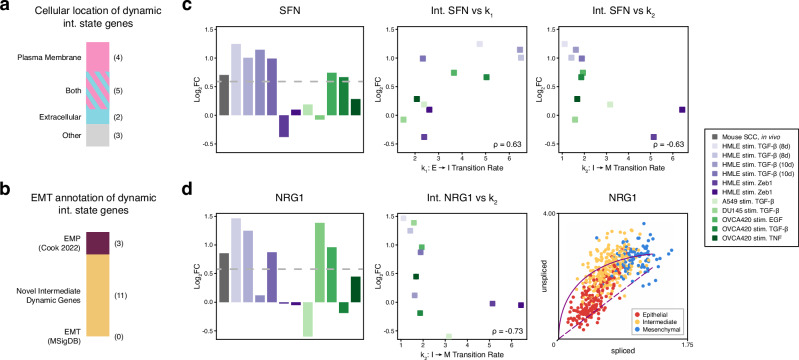
Genes influencing EMT intermediate state dynamics are identified via consensus analysis of expression, regulation, and parameter inference. **a** Annotation of predicted dynamic intermediate state genes by cellular location. **b** Annotation of predicted dynamic intermediate state genes by EMT marker gene sources. **c** *SFN* is a predicted dynamic intermediate state gene, with its differential expression in multiple EMT intermediate states and significant correlations with model parameters *k*_1_ and *k*_2_. **d** *NRG1* is another predicted dynamic intermediate state gene, with its differential expression in multiple EMT intermediate states, significant negative correlation with model parameter *k*_2_, and differential regulation in EMT intermediate states via RNA velocity.

To identify genes influencing intermediate EMT dynamics, we studied associations between intermediate EMT genes and fitted parameters of the mathematical model. A gene’s positive correlation with *k*_1_ indicates faster transition *E* → *I*, while a negative correlation with *k*_2_ means a slower transition *I* → *M*; either correlation suggests that the gene is associated with a more persistent intermediate state. Genes with significant Spearman’s correlation were compared with differential expression and differential velocity genes in intermediate states, and those supported by multiple lines of evidence were consolidated into a consensus gene list of 14 genes (Supplementary Table 3 and Supplementary Fig. 11). The majority of intermediate EMT dynamics genes were located at the plasma membrane or in the extracellular region (Fig. 5a). Of the 14 predicted intermediate EMT dynamics genes, three were identified in a prior EMP study^46^ (Fig. 5b), consistent with the conceptual overlap between intermediate EMT dynamics and EMP. Notably, there is no overlap between intermediate EMT dynamics genes and those from hallmark EMT (mesenchymal) genes, demonstrating that our proposed gene set is novel and distinct from previous EMT gene sets.

Two predicted EMT dynamics genes had the strongest support (three lines of evidence each; Supplementary Table 3). *NRG1* was the only gene identified in all three analyses, while *SFN* was the only gene with intermediate EMT differential expression and significant correlations with both *k*_1_ and *k*_2_ transition rates. Stratifin (*SFN*) was positively correlated with *k*_1_ (*E* → *I*) and negatively correlated with *k*_2_ (*I* → *M*) across cancer samples (Fig. 5c), suggesting that it stabilizes the intermediate EMT state. Although RNA velocity for *SFN* was not captured due to insufficient counts, it was differentially expressed in intermediate states. Neuregulin 1 (*NRG1*) was negatively correlated with *k*_2_ (*I* → *M*), suggesting it slows the exit from the intermediate state (Fig. 5d), and *NRG1* was also significant in intermediate EMT differential expression and velocity (Supplementary Fig. 8).

Consensus gene analysis predicts that *SFN* promotes transitions from an epithelial state to the metastatic intermediate EMT state. This prediction helps to reconcile literature, which reports both epithelial and pro-EMT roles for *SFN*. Named for its expression in stratified epithelial cells^51^, *SFN* can be secreted and is found in extracellular vesicles^73^. Recombinant *SFN* treatment has been shown to significantly enhance extracellular matrix degradation in human dermal fibroblasts in vitro^74^. Despite its epithelial association, *SFN* knockdown in in vitro models has led to reduced mesenchymal marker expression in cervical cancer cells^52^ and decreased cell migration in other carcinomas^53,54,75^. In vivo, *SFN* knockdown suppressed tumor formation and metastasis in lung adenocarcinoma models^76^. Clinically, *SFN* is linked to poor prognosis, including advanced tumor stages in lung adenocarcinoma and hepatocellular carcinoma^53,77^, as well as lower survival rates in pancreatic ductal adenocarcinoma^78^ and head and neck squamous cell carcinoma^79^. Our findings suggest that *SFN* promotes intermediate EMT dynamics, potentially explaining its dual role in epithelial cells while facilitating EMT.

Consensus gene analysis also identified *NRG1* as playing a pivotal role in intermediate EMT state dynamics, as the sole gene that was significant in intermediate expression, regulation, and modeled dynamics. A member of the epidermal growth factor (EGF) family^71^, *NRG1* activates *ERBB2* (*HER2*) and *ERBB3* (*HER3*)^80^. *NRG1* isoforms can be found in the plasma membrane or secreted^81^, and it binds integrins including *ITGA6*:*ITGB4* and *ITGAV*:*ITGB3*^82^. In vivo, *NRG1* suppression reduces tumor growth and metastasis in hepatocellular carcinoma^83^. Clinically, *NRG1* overexpression correlated with poor outcomes, including lymph node metastasis, in gastric cancer^84^. Notably, *NRG1* has been found to promote partial EMT in cultured patient *HER2*-positive breast cancer^85^. While *NRG1* has been mostly described to drive EMT in epithelial cells, *NRG1* stimulation on mesenchymal cells that already underwent EMT has been shown to instead induce epithelial gene expression in esophageal adenocarcinoma^86^. Taken together, our analyses along with literature suggest that *NRG1* is a marker of highly plastic intermediate state cells during EMT.

### *SFN* is a marker of intermediate state EMT in independently analyzed MCF10A cells

**Fig. 6:**
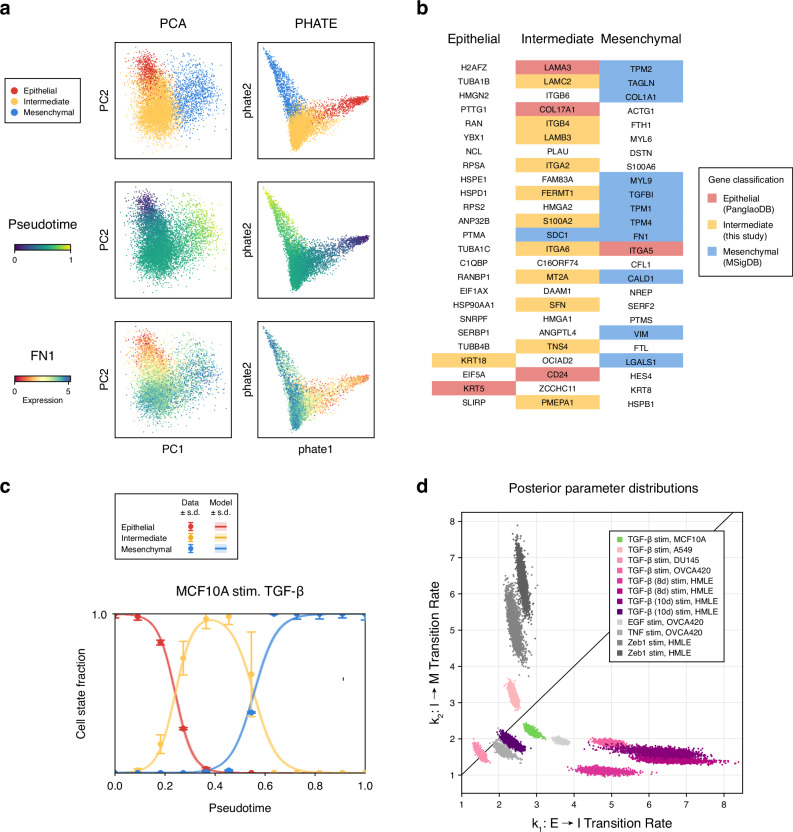
*SFN* is identified as a marker of intermediate EMT in an analysis of dose-dependent EMT. **a** MCF10A cells were analyzed separately and exhibit a linear trajectory across EMT states. **b** For each MCF10A cell state, the top 25 differentially expressed genes colored by gene set annotation. **c** Model fit following parameter inference: data vs. trajectory simulations, with simulation parameters sampled from the posterior distribution. **d** Comparison of posterior parameter distributions, with the MCF10A sample highlighted in green. Other distributions are replicated from Fig. 4d, shown in different colors here to highlight stimulation by TGF-*β*.

To assess predicted intermediate EMT genes, we analyzed a dataset of EMT under different experimental conditions and in a different cell line: the dose-dependent TGF-*β* stimulation of MCF10A breast cells^87^. Similar to previous analyses, scRNA-seq data was clustered, and canonical markers were used to identify epithelial, intermediate, and mesenchymal states (Fig. 6a). Differential expression by cell state showed strong agreement with our predictions, with 11 of the top 25 intermediate state genes in this sample overlapping with our predicted intermediate EMT genes (Fig. 6b), notably including *SFN*. These results highlight that shared EMT intermediate state features can be found across diverse biological and experimental conditions, with independent evidence corroborating one of the top genes associated with intermediate EMT.

To assess EMT dynamics in these MCF10A cells, we applied the mathematical model using the same analytical pipeline (Fig. 6c). While dose-dependent EMT does not follow a true temporal progression, single-cell heterogeneity across TGF-*β* doses was evident. We used a pseudotime axis to represent a continuum of EMT states, capturing EMT transitions with different TGF-*β* doses. The posterior parameter distribution lies in the region where *k*_1_ ≥ *k*_2_, consistent with EMT dynamics induced by TGF-*β* in other cell types (Fig. 6d). Across different cancer types, we see that mammary (MCF10A and HMLE) and ovarian (OVCA420) cells stimulated with TGF-*β* generally exhibit *k*_1_ > *k*_2_ dynamics, favoring stabilization of the intermediate state. In contrast, lung (A549) and prostate (DU145) cells stimulated with TGF-*β* show balanced rates of entry and exit from the intermediate state, with *k*_1_ ≈ *k*_2_. The similarity in transition dynamics between mammary and ovarian cells is notable, given the shared genetic and microenvironmental factors during oncogenesis and tumor progression^88^.

## Discussion

Here, we characterized intermediate EMT states and identified genes involved in dynamic transitions between states. Multiple lines of evidence suggest EMT intermediate states are the most cancer stem-like and exhibit the highest metastatic potential^43,89–92^. Our analysis predicted intermediate state genes in agreement with recent work, such as *ITGB4* and *LAMB3*^93^, as well as novel EMT intermediate genes, such as *SFN* and *NRG1*. While there are many paths of EMT, our comparison across different cell types and stimuli revealed common markers for intermediate states and highlighted the role many of these genes have in extracellular remodeling.

EMT is heterogeneous^45^. Multiple transcription factors can initiate EMT^14^ and act in complex and nonlinear ways, both alone^94^ or in combination^95^. Future work could shed more light on EMT intermediate state transitions by broadening the scope of EMT-inducing factors^96^. Additional factors contributing to EMT complexity, including subtypes and intermediate states, are hysteresis during the reverse mesenchymal-epithelial transition, differences in cell types or stimuli, and state transitions driven by intrinsic or extrinsic noise. Whereas EMT is most frequently modeled via gene regulatory networks, here we modeled the population dynamics to study cell state heterogeneity and its effects on EMT path variation. In doing so, we assumed a monostable landscape, whereas in reality multiple stable steady states exist^9^. Some of the gene expression heterogeneity underlying these multiple states is likely collapsed by this approach, but in doing so we can identify consensus genes marking for properties of EMT states across different conditions. Our model can be adapted in the future to consider multiple intermediate states and more complex (e.g., convergent/divergent) EMT paths.

Summarizing complex data across conditions to find consensus requires simplifying assumptions. To compare gene expression across datasets, we used log-fold changes and rank-based comparisons, similar to other recent work^97^. Doing so relies on the accurate quantification of cell states, which is not guaranteed, and can obscure single-cell resolution information by taking pseudo-bulk measurements. While we sought to standardize data analysis pipelines as far as possible, scRNA-seq data analysis relies on certain parameter choices. While clustering cells, we sought fewer clusters (lower resolution) where supported, to reduce overfitting cell states. Clustering-based cell state definitions differ between studies: ref. ^43^ identified four EMT states, whereas ref. ^98^ later identified five in the same dataset. Similarly, ref. ^99^ identified five states in the ref. ^41^ dataset, aligning with experimental timepoints. Each approach reveals distinct aspects of EMT; we take the perspective of using pseudotime to infer EMT dynamics over a 3–4 cell state space across the EMT spectrum. Trajectory inference relies on accurate choice of root cells and the sufficiency of the similarity metric used. RNA velocity analysis is limited by the ratio of spliced to unspliced counts, typically around 75–85% spliced to 15–25% unspliced^69^. This abundance limitation affects genes with low or no unspliced counts, such as *SFN* in our study, where RNA velocity analysis could not be performed due to a lack of unspliced counts. This abundance limitation could be addressed by experimental methods targeting dynamics, such as RNA metabolic labeling^36^. We applied a standardized pipeline to each dataset while preserving dataset-specific parameters. Although integration could reveal shared EMT features, differences in experimental design, single-cell platforms, and study conditions complicate the distinction between biological variation and technical batch effects. To maintain dataset-specific nuances, we prioritized a comparative analysis of differential expression and dynamic model parameters, though future studies incorporating batch correction may help determine whether intermediate EMT states share a universal transcriptional signature.

Mathematical modeling and parameter inference with single-cell data allow us to investigate the genes and pathways associated with dynamic transitions between states rather than the cell states themselves—transitions which are strongly relevant to epithelial–mesenchymal plasticity^100^. EMP, exemplary of cell state plasticity, has been shown to play decisive roles in tumorigenesis and cancer progression^101,102^. This property can assist tumors in developing powerful “generalist” phenotypes as they evolve^103^. The mathematical model with which we study EMT population dynamics is phenomenological: capturing the rates of entry/exit between EMT states without transcriptional information or feedback signaling. It does not incorporate additional complexities such as reverse transitions or stochasticity. We have used external information from the biological properties of EMT to construct our mathematical model, and not obtained it purely from the cell dynamics observed in the data^35^. Nonetheless, to compare relative transition rates, a simple three-compartment model seems reasonable to describe most conditions analyzed and fits both the inferred cell states (clusters) and the pseudotemporal dynamics during EMT. Incorporating cell proliferation and death could refine EMT modeling, but doing so presents challenges in parameter identifiability. Our approach prioritized a parsimonious model, using normalized cell proportions to implicitly account for differences in cell numbers, though this does not explicitly capture variations in cell survival. Future extensions integrating proliferation and death rates, potentially constrained by lineage tracing or live-cell imaging, could provide a more comprehensive understanding of EMT dynamics and intermediate state persistence. Additional future work could include combining cell population dynamics with a transcriptional EMT network^9^ to investigate the role of cell–cell communication^104^ on the population dynamics of EMT—though additional data may be required for the transcriptional dynamics of such a model to avoid double dipping^105^.

Canonical EMT states are defined by morphological features: epithelial cells adhere to each other with apical–basal polarity; mesenchymal cells are spindle-shaped, migratory, and lack cell–cell adhesion^106^. These morphological/adhesive properties cannot be fully captured by sequencing data alone. Moreover, multiple EMT gene lists (typically focusing on mesenchymal traits) have been proposed, with varying levels of agreement^45,46,89,107,108^. This variability in consensus genes also applies to epithelial genes, which can show tissue-specific heterogeneity. No single gene list can do justice to the heterogeneous paths of EMT, yet as we have shown, distinctive dynamic properties of EMT intermediate states can be captured by marker genes. Although our study focused on cancer-related EMT, the in vitro stimuli such as TGF-*β* also apply to healthy EMT, suggesting potential relevance beyond cancer. Nevertheless, the heterogeneity observed among tumor cell lines underscores the need to investigate EMT in wound healing and tissue regeneration to determine whether the identified marker genes and intermediate states are conserved in the context of non-malignant EMT.

Genes predicted here as candidate markers of intermediate state EMT genes may serve as biomarkers of cells likely to metastasize and could be tested as predictors of clinical progression. In addition, such genes may mark for high-risk tumor cells prone to metastasis or recurrence, given the high metastatic potential of EMT intermediate state cells^16,21,92,109^. More broadly, this study has shed new light on the plasticity of the EMT landscape and how it shapes the cell state transitions underlying cancer metastasis.

## Methods

### scRNA-seq data sources

In this study, we conducted an integrated analysis of several single-cell RNA sequencing (scRNA-seq) datasets in the public domain. We included datasets from Pastushenko et al.^43^ (GEO accession GSE110357); van Dijk et al.^110^ (GSE114397); Cook and Vanderhyden^41^ (GSE147405); and Panchy et al.^87^ (GSE213753). For data from Cook and Vanderhyden^41^, samples collected after the removal of the EMT stimulus were not included. For data from Panchy et al.^87^, unstimulated cells were not included.

### scRNA-seq sequence alignment

Data from ref. ^41^ were re-aligned to obtain spliced and unspliced read counts for RNA velocity analysis below. Raw sequence files (accession SRP253729) were downloaded from the NIH Sequence Read Archive using the SRA Toolkit^111^ and converted from SRA to FASTQ files using fasterq-dump. Python package cutadapt was used to trim the barcode sequences to 26 base pairs^112^. The splici (spliced+intron) index was constructed using the GRCh38 human reference genome with Python package salmon^113^. Sequence pseudoalignment was performed with salmon alevin-fry. Barcode demultiplexing was carried out using the R package MULTIseq^114^. Contaminant cells in the OVCA420 samples were removed as noted by the original authors.

### scRNA-seq data preprocessing and normalization

All scRNA-seq data were processed and analyzed using Scanpy^115^. Cells with fewer than 200 genes and genes expressed in fewer than three cells were filtered out. Cells with high mitochondrial percentages or disproportionately high total read counts were excluded based on dataset-specific cutoffs (Supp. Table 1). In HMLE samples stimulated with TGF-*β*, cells with disproportionately high ribosomal percentages were filtered out (<1% of cells). Counts were normalized to 10,000 and *log*(*x* + 1) transformed. Batch correction for samples from^41^ was performed using ComBat in Scanpy^116^. Cell cycle effects, which significantly impacted clustering by EMT state identity, were regressed out^117,118^, similar to the original analyses. Additional preprocessing included regressing out total counts and percent mitochondrial counts per cell, scaling counts to uniform variance, and selecting highly variable genes for downstream analysis.

### Cell clustering and scoring by EMT status

Principal component analysis (PCA) was performed, and the top 15 components were used to construct a nearest-neighbor graph. Based on this graph, cell clustering was conducted using the Leiden algorithm^119^ with dataset-specific Leiden resolutions (Supplementary Table 1). Silhouette scores were computed for Leiden resolutions ranging from 0.3 to 1.0 in 0.05 increments using silhouette_score from scikit-learn^120^. Differentially expressed genes for each cell cluster were identified using the Wilcoxon rank-sum test with Benjamini–Hochberg correction. Cell clusters were visualized in two dimensions using UMAP and PHATE^121,122^.

To infer the EMT status of single cells based on a set of EMT marker genes, an “EMTscore” was created using the UCell scoring method^42^ with the Hallmark EMT gene set from the Molecular Signatures Database (MSigDB)^39,123^. UCell calculates single-cell gene expression scores from a gene set using a rank-based approach, which we found to effectively quantify EMT across disparate tumor types and experimental conditions. Genes were input into UCell as filtered and normalized counts.

### Identifying shared EMT intermediate state genes

Genes were included in the intermediate state analysis if they were differentially expressed (DE) in an intermediate state with a Benjamini–Hochberg adjusted Wilcoxon rank-sum *P* value of *P* < 0.01, up to a maximum of 500 genes per sample. To account for the complexities of comparing gene expression across different datasets and conditions (e.g., batch effects, instrumentation, sequencing depth), we calculated log_2_ fold change (log_2_FC) values of intermediate state genes in Scanpy, following the notation of Moses et al.^124^:

1 $$\begin{array}{ll}{\log }_{2}\,{\rm{FC}}\, {\rm{of}}\, {\rm{gene}}\,g={\log }_{2}\left(\exp \left(\frac{1}{{n}_{1}}\sum\limits_{i\in {G}_{1}}{Y}_{ig}\right)-1+\epsilon \right)\\\qquad\qquad\qquad\qquad-{\log }_{2}\left(\exp \left(\frac{1}{{n}_{2}}\sum\limits_{i\in {G}_{2}}{Y}_{ig}\right)-1+\epsilon \right)\end{array}$$

where *G*_1_ is the focal group of cells of size *n*_1_, *G*_2_ is the comparison group with *n*_2_ cells, and *Y*_*ig*_ denotes the log-normalized counts of gene *g* in cell *i*. The pseudocount *ϵ* = 10^−9^ is added to avoid division by zero^124^. Genes were selected as intermediate state-associated if they met the following criteria: (i) a log_2_FC ≥0.58 (1.5-fold change) in at least five samples, and (ii) at least two of these samples were from experiments not performed on HMLE cells. Gene set enrichment analysis (GSEA) was performed on identified intermediate state genes^123^.

### Trajectory inference and EMT subpopulation dynamics

Diffusion pseudotime (DPT) was used for trajectory analysis^125^. Root nodes were chosen as the epithelial cells with extreme coordinates on a diffusion map. Pseudotime was calculated five times with different epithelial root nodes, and the median values were assigned to each cell, with the standard deviation indicating pseudotime variation. This approach minimized the impact of root node selection on pseudotime calculation. Pseudotime values range from 0 (epithelial) to 1 (mesenchymal). This range was divided into 15 bins (12 for Pastushenko et al.^43^ due to fewer cells), and cell counts were calculated for each cluster (epithelial, intermediate, and mesenchymal) for each bin. The counts per bin were converted into cell population proportions.

### RNA velocity analysis

RNA velocity analysis was conducted in Python using the package scVelo in dynamical mode on highly variable genes^70^. Each sample was analyzed individually. Differential velocity (DV) was assessed using the rank_dynamical_genes function on clusters. Genes with a DV score above 0.25 were retained as DV genes. To ensure monotonic transitions, genes with Spearman correlation coefficients below 0.5 were excluded. In addition, DV genes with poor dynamical model fits were filtered out. Ultimately, we retained DV genes that were upregulated in the majority of cancer samples, designating them as shared upregulated velocity genes across EMT.

### A mathematical model of EMT dynamics

We developed a mathematical model of the dynamics of EMT described by ordinary differential equations (ODEs). Specifically, we sought to describe the cell state transitions during EMT, from the epithelial (*E*) to intermediate (*I*) state or states, and then to the mesenchymal (*M*) state. While EMT systems may also exhibit direct transitions (*E* → *M*) and reverse transitions, our data specifically investigate forward EMT and do not exhibit strong evidence for direct transitions.

The population dynamics of *E*, *I*, and *M* are described by:

2 $$\begin{array}{lll}\frac{dE}{dt}=-{k}_{1}EI\\ \frac{dI}{dt}={k}_{1}EI-{k}_{2}IM\\ \frac{dM}{dt}={k}_{2}IM\end{array}$$

where *k*_1_ denotes the transition rate from *E* to *I*, and *k*_2_ denotes the transition rate from *I* to *M*. We consider second-order transitions, meaning both the initial and final states influence the transition rate to the final state. In cases where more than one intermediate state exists, the model can be extended using the same framework (Supplementary Fig. 9A).

### Parameter inference of cell population dynamics over pseudotime

We sought to infer the rates of EMT using Bayesian parameter inference with the Turing.jl package in Julia^126–128^. The input data for each model consists of the cell state dynamics over pseudotime. To focus on relevant dynamics, we excluded periods where all cells remained in the epithelial state. Timepoints along pseudotime were normalized to a range of *t* ∈ [0, 10], facilitating direct comparison of EMT trajectories across samples. For each sample with one intermediate state, we fit three parameters: *k*_1_, *k*_2_, and the observational noise parameter *σ*. For the in vivo sample with two intermediate states, we fit four parameters: *k*_1_, *k*_2_, *k*_3_, and *σ*.

Letting *f* represent the numerical solution to the ODE model and *y*_0_ the initial conditions, we performed parameter inference as follows:

3 $$\begin{array}{lll}{\theta }_{{k}_{i}}& \sim &{\mathcal{N}}(4,1)\\ \sigma & \sim &\,{\text{Inv}}\!-\!{\text{Gamma}}\,(3,1)\\ \widehat{y}(t)&=&f({y}_{0},t;\theta )\\ y(t)& \sim &{\mathcal{N}}(\widehat{y}(t),\sigma )\end{array}$$

where $\theta =({\theta }_{{k}_{i}},\sigma )$ gives the prior parameter distribution, and *y*(*t*) defines the likelihood function in terms of ODE model simulation ($\widehat{y}(t)$) for transition rate parameters ${\theta }_{{k}_{i}}$ and noise parameter *σ*.

The posterior parameter distribution was estimated via Markov chain Monte Carlo (MCMC) simulations using the No-U-Turn Sampler (NUTS)^129^. MCMC chains were each run for 1000 iterations following 250 warmup iterations to ensure convergence. Fitted trajectories were visualized by solving the model using 300 joint parameter sets of *k*_*n*_, randomly selected from the posterior distribution for each sample, and plotting the mean and standard deviation of the resulting trajectories.

### Comparative analysis of EMT intermediate state-associated genes

We identified genes associated with EMT transition rates by analyzing correlations between model-inferred posterior parameters and gene expression. For each transition rate parameter *k*_*n*_, we used its maximum a posteriori value for each sample and examined pairwise correlations with the log_2_FC expression of 145 genes, each present in at least 5 samples with an intermediate state log_2_FC ≥ 0.2. Genes with a Spearman’s rank correlation coefficient of *ρ* > ∣0.6∣ (*P* < 0.05) were considered associated with, and potential influencers of, transitions into or out of EMT states.

To specifically identify genes linked to EMT intermediate state dynamics, we focused on genes positively correlated with *k*_1_ (faster *E* → *I*) and negatively correlated with *k*_2_ (slower *I* → *M*). Genes meeting both correlation criteria were included, as well as those showing either correlation as well as differential expression or differential velocity in the intermediate state. Cellular location annotations were performed using DAVID^72,130^ and PANTHER^131^.

## Supplementary information

Supplementary information

Supplementary Data 2

## Data Availability

All scRNA-seq data used in this study are publicly available on the Gene Expression Omnibus (GEO). We included datasets from Pastushenko et al.^43^ (accession GSE110357); van Dijk et al.^110^ (GSE114397); Cook and Vanderhyden^41^ (GSE147405); and Panchy et al.^87^ (GSE213753). Raw sequence files from Cook and Vanderhyden^41^ were downloaded from the NIH Sequence Read Archive (accession SRP253729). Supplementary Tables and Figures are provided as a separate document. Supplementary datasets provided are: gene expression plots of all 32 identified EMT intermediate state genes (Supplementary Data 1, available at: https://github.com/maclean-lab/dynamicEMT-genes under 3–DE genes) and GSEA results (Supplementary Data 2).

## Code availability

All code and data analysis associated with this study are released under an MIT license, available on GitHub: https://github.com/maclean-lab/dynamicEMT-genes.
