# Supplementary material for: Modeling the dynamics of EMT reveals genes associated with pan-cancer intermediate states and plasticity
Source: NPJ Syst Biol Appl. 2025 Apr 10;11:31. doi: 10.1038/s41540-025-00512-2 (PMC11986130; doi:10.1038/s41540-025-00512-2)
Supplement: Supplementary file 1 — Supplementary information [file 41540_2025_512_MOESM1_ESM.pdf]

Supplementary Information

Modeling the dynamics of EMT reveals genes associated with pan-cancer intermediate states and plasticity

MeiLu McDermott[1], Riddhee Mehta[1], Evanthia T. Roussos Torres[2] & Adam L. MacLean[1,*]

[1]Department of Quantitative and Computational Biology, Dornsife College of Letters, Arts and Sciences, University of Southern California, Los Angeles, CA 90089, USA
[2]Department of Medicine, Division of Medical Oncology, Keck School of Medicine, Norris Comprehensive Cancer Center, University of Southern California, Los Angeles, CA 90033, USA
[*]Correspondence: macleana@usc.edu (A.L.M.)

Contents

SUPPLEMENTARY TABLES 1

SUPPLEMENTARY FIGURES 4

SUPPLEMENTARY TABLES

Sample	Total counts threshold	Percent mitochondrial counts threshold	Percent ribosomal counts threshold	Leiden resolution
Mouse SCC, *in vivo*	100,000	12%	–	0.4
HMLE stim. TGF-β (8d)	10,000	12%	12%	0.4
HMLE stim. TGF-β (8d)	10,000	12%	12%	0.4
HMLE stim. TGF-β (10d)	10,000	12%	12%	0.35
HMLE stim. TGF-β (10d)	10,000	12%	12%	0.4
HMLE stim. Zeb1	10,000	15%	–	0.75
HMLE stim. Zeb1	10,000	15%	–	0.7
A549 stim. TGF-β	40,000	8%	–	0.4
DU145 stim. TGF-β	50,000	8%	–	0.4
OVCA420 stim. EGF	35,000	10%	–	0.55
OVCA420 stim. TGF-β	35,000	10%	–	0.5
OVCA420 stim. TNF	40,000	10%	–	0.45
MCF10A stim. TGF-β	35,000	7%	–	0.2

Supplementary Table 1: *scRNA-seq processing parameters for each dataset. A standardized pipeline was applied across all datasets.*

Sample	$k1$,<br>Transition Rate $E \rightarrow I$	$k2$,<br>Transition Rate $I \rightarrow M$
HMLE stim. TGF-β (8d)	4.77	1.11
HMLE stim. TGF-β (8d)	6.45	1.40
HMLE stim. TGF-β (10d)	6.52	1.61
HMLE stim. TGF-β (10d)	2.31	1.87
HMLE stim. Zeb1	2.36	5.06
HMLE stim. Zeb1	2.60	6.40
A549 stim. TGF-β	2.38	3.16
DU145 stim. TGF-β	1.50	1.57
OVCA420 stim. EGF	3.65	1.93
OVCA420 stim. TGF-β	5.06	1.86
OVCA420 stim. TNF	2.07	1.68
MCF10A stim. TGF-β	2.88	2.18

Sample	$k1$,<br>Transition Rate<br>$E \rightarrow I1$	$k2$,<br>Transition Rate<br>$I1 \rightarrow I2$	$k3$,<br>Transition Rate<br>$I2 \rightarrow M$
Mouse SCC, *in vivo*	1.99	3.88	1.79

Supplementary Table 2: *Fitted mathematical model parameters k_n for each scRNA-seq dataset. The in vivo mouse SCC dataset has two intermediate states with three k_n parameters. All other in vitro datasets have one intermediate state with two k_n parameters.*

Gene	Int. DE	Int. DV	k_1 pos corr	k_2 neg corr
SFN	●	○	●	●
NRG1	●	●	○	●
ITGB4	●	●	○	○
ITGA6	●	○	○	●
CBFB	●	○	●	○
FAM111A	●	○	○	●
LINC01503	●	○	○	●
PLEK2	●	○	○	●
IL4R	●	○	○	●
STK17A	○	○	●	●
CENPW	○	○	●	●
KRT18	○	○	●	●
GJB3	○	○	●	●
FHOD3	○	○	●	●

Supplementary Table 3: *Genes influencing intermediate EMT dynamics. DE denotes genes differentially expressed in intermediate states, and DV denotes genes with differential velocity in intermediate states. A positive k_1 correlation indicates faster $E \rightarrow I$ transition, while a negative k_2 correlation indicates slower $I \rightarrow M$ transition.*

SUPPLEMENTARY FIGURES

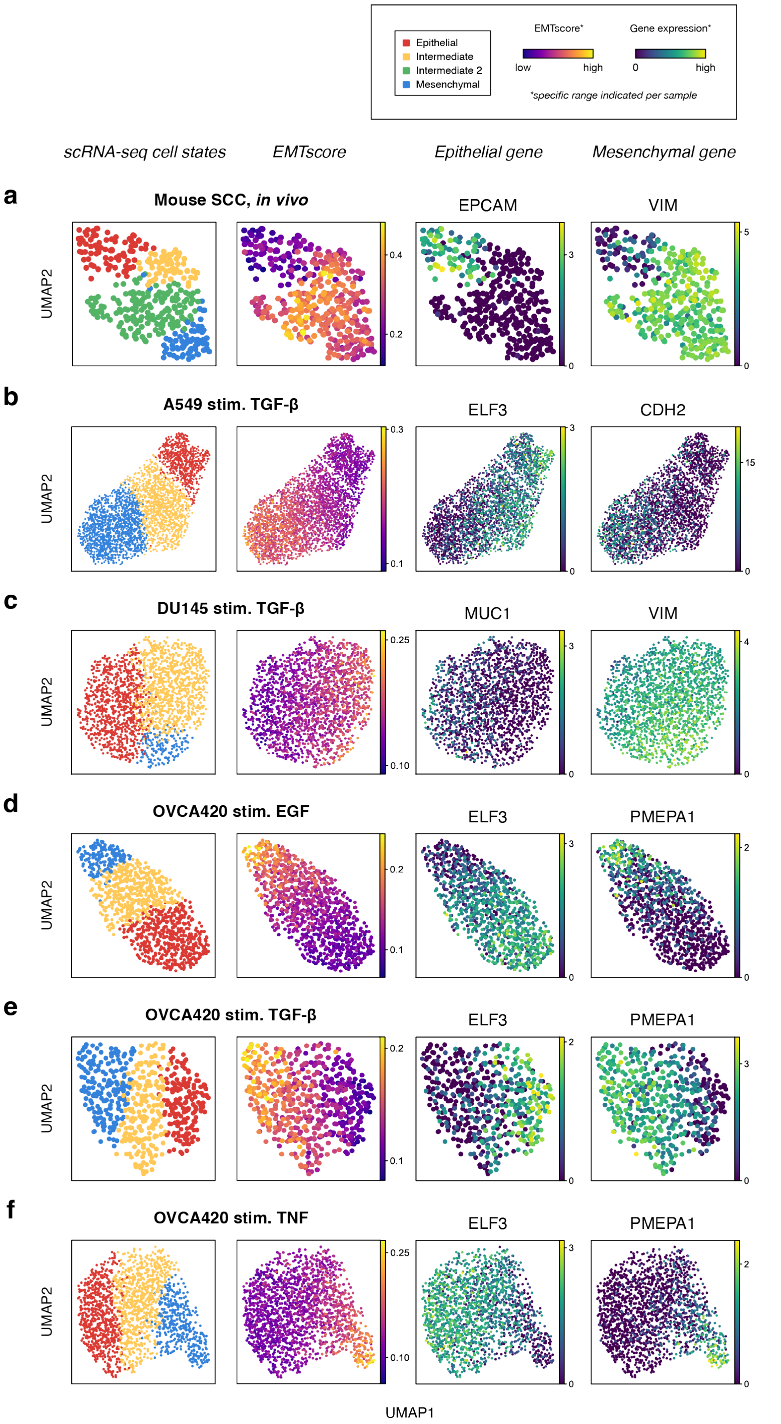

Supplementary Figure 1: ***scRNA-seq data analysis of Pastushenko et al., 2018 and Cook and Vanderhyden, 2020. a-f.*** *Cell states were identified via Leiden clustering, EMT scores were calculated with UCell, and representative epithelial and mesenchymal genes are depicted.*

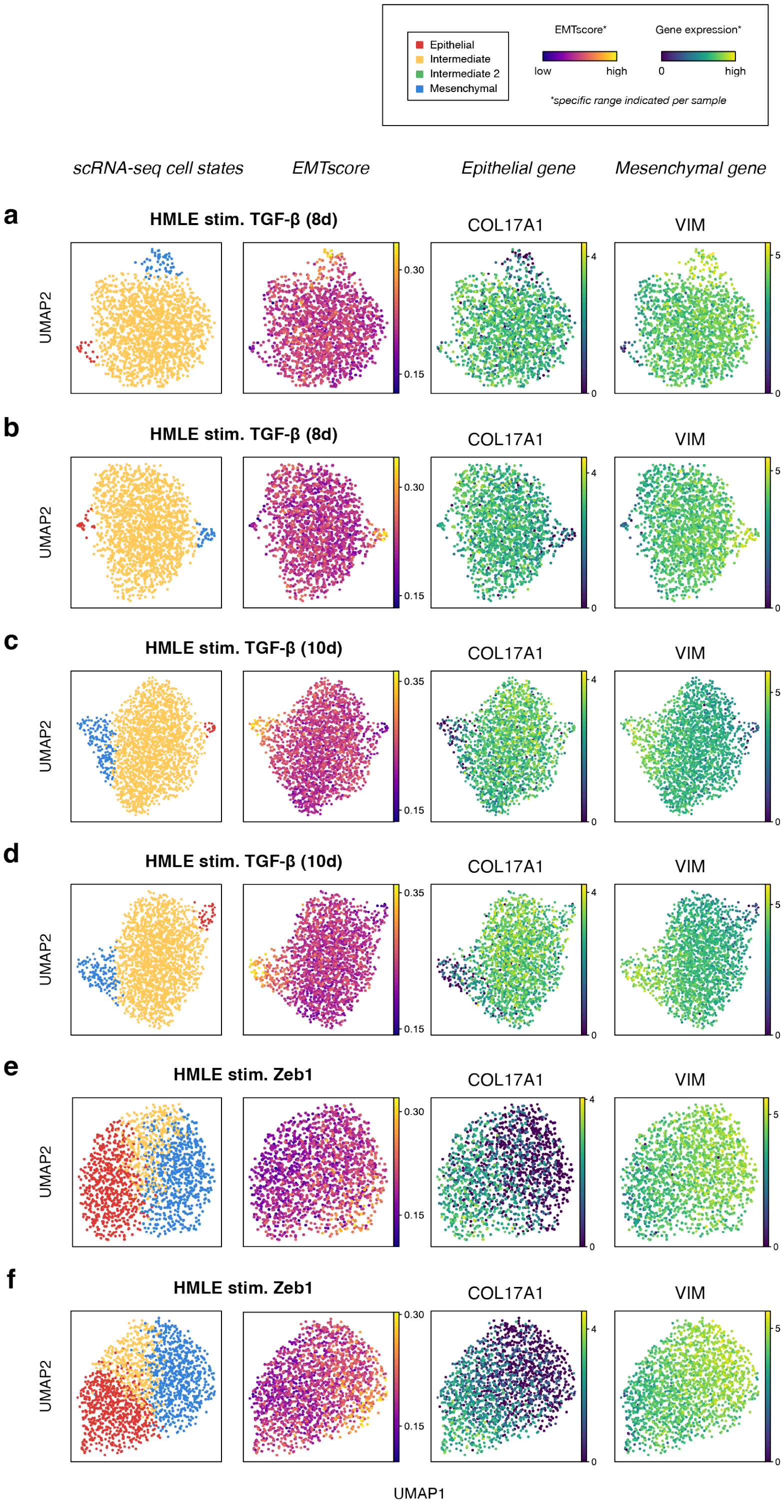

Supplementary Figure 2: ***scRNA-seq data analysis of van Dijk et al., 2018. a-f.*** *Cell states were identified via Leiden clustering, EMT scores were calculated with UCell, and representative epithelial and mesenchymal genes are depicted.*

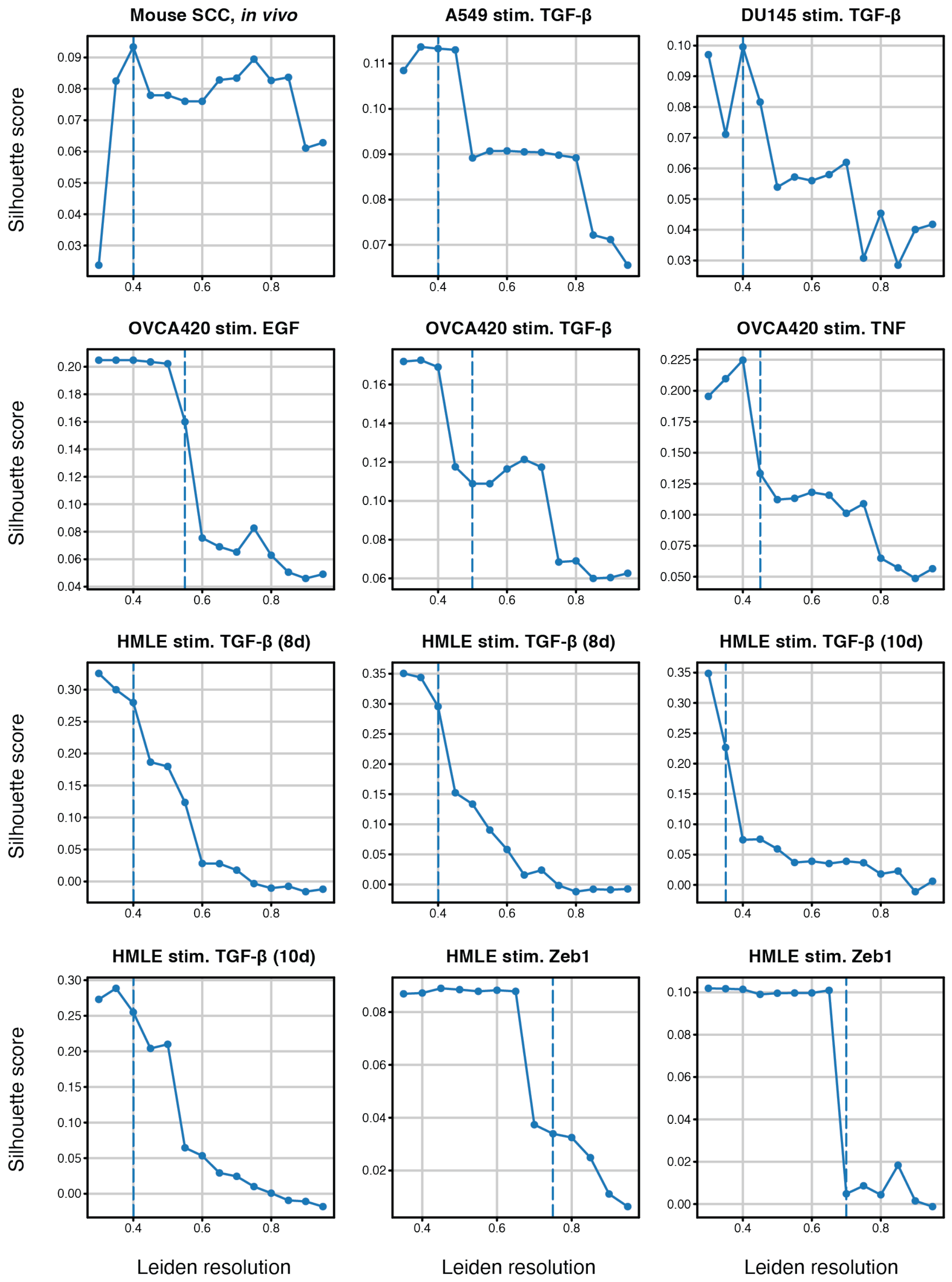

Supplementary Figure 3: ***Silhouette scores assess Leiden algorithm resolution parameter for each dataset.*** *Dashed lines indicate the selected resolution. Higher silhouette scores denote more robust clustering by reflecting greater intra-cluster similarity relative to inter-cluster dissimilarity. Lower Leiden resolution values produce fewer clusters, while higher resolutions generate more. The chosen Leiden resolution aims to balance the trade-off between cluster number and robustness.*

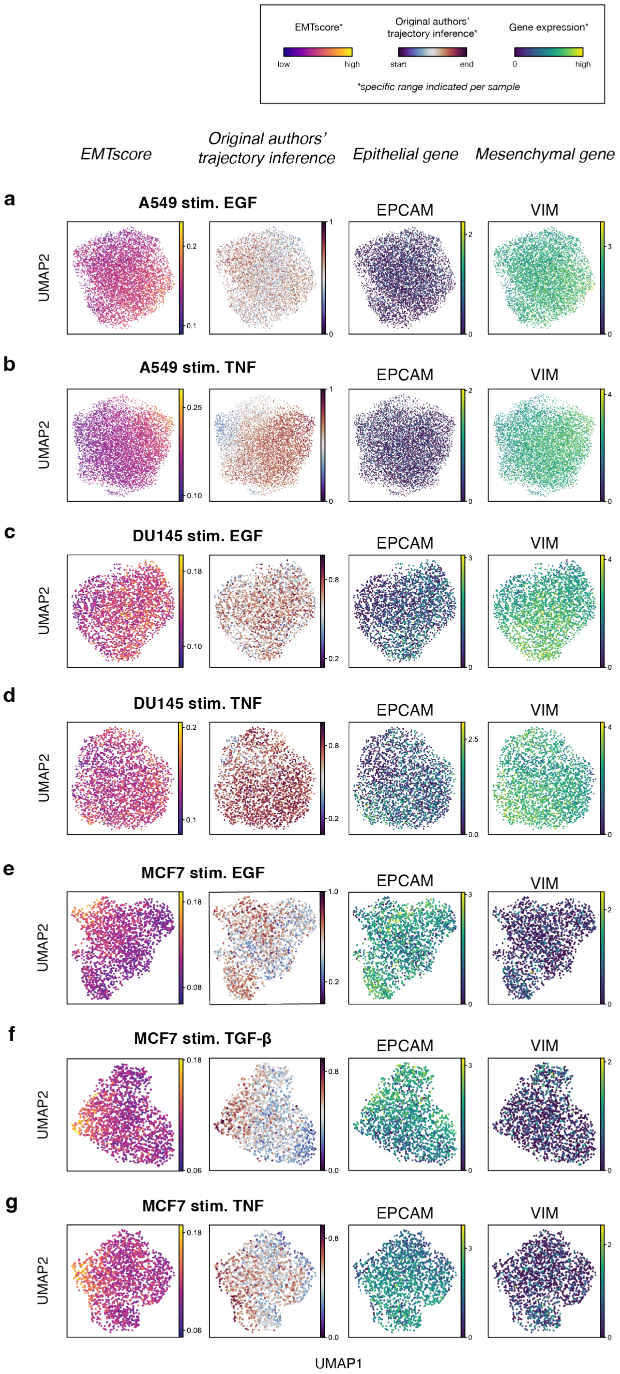

Supplementary Figure 4: ***scRNA-seq data analysis of Cook and Vanderhyden, 2020 samples that did not exhibit a clear EMT and were excluded from the main analysis. a-g.*** *EMT scores were calculated using UCell, and trajectory inference was obtained from the original publication. Representative epithelial and mesenchymal genes (EPCAM and VIM) are shown but did not align with distinct EMT cell states. The lack of EMT trajectory exemplified through both EMT scores and pseudotime supports the lack of complete EMT in these samples.*

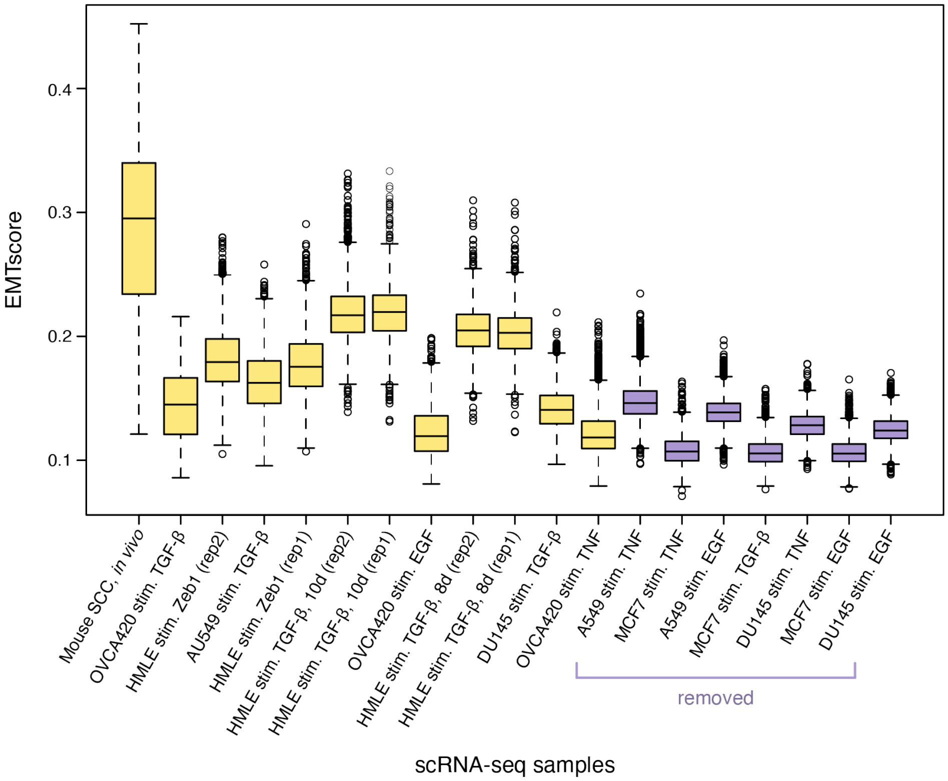

Supplementary Figure 5: ***EMTscore distributions for all scRNA-seq datasets, both included and excluded from the main analysis.*** *Mean and inter-quartile range of each dataset are shown, and the distributions sorted by range from highest to lowest. Datasets were excluded if they did not exhibit EMT according to EMT marker genes (see Supp. Fig. 4), and the small EMTscore ranges reinforce their lack of complete EMT.*

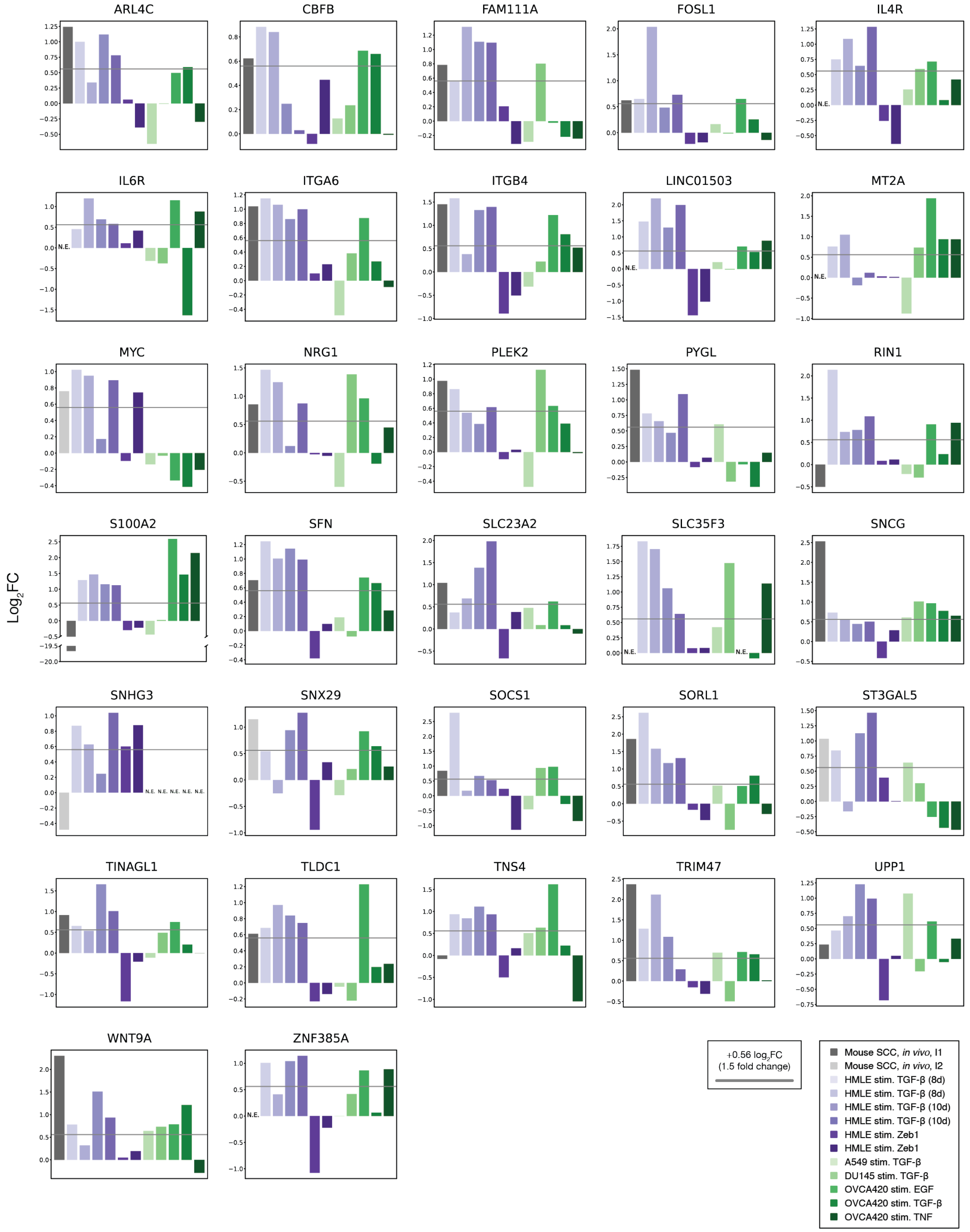

Supplementary Figure 6: ***Genes associated with the intermediate EMT state.*** *Genes are identified by log_2FC upregulation in the intermediate state across scRNA-seq samples.*

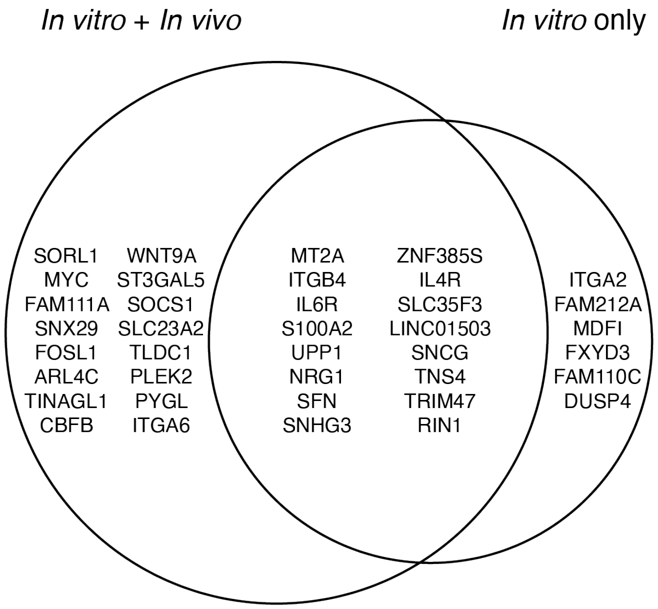

Supplementary Figure 7: ***Assessment of tumor microenvironment (TME) influence on intermediate EMT gene expression.*** *The left segment of the Venn diagram includes our inferred intermediate EMT genes from in vitro and in vivo experiments, capturing TME contributions. The right segment only includes in vitro data, excluding TME influences. This comparison highlights greater gene expression complexity when in vivo data are included, underscoring the modulatory role of the TME.*

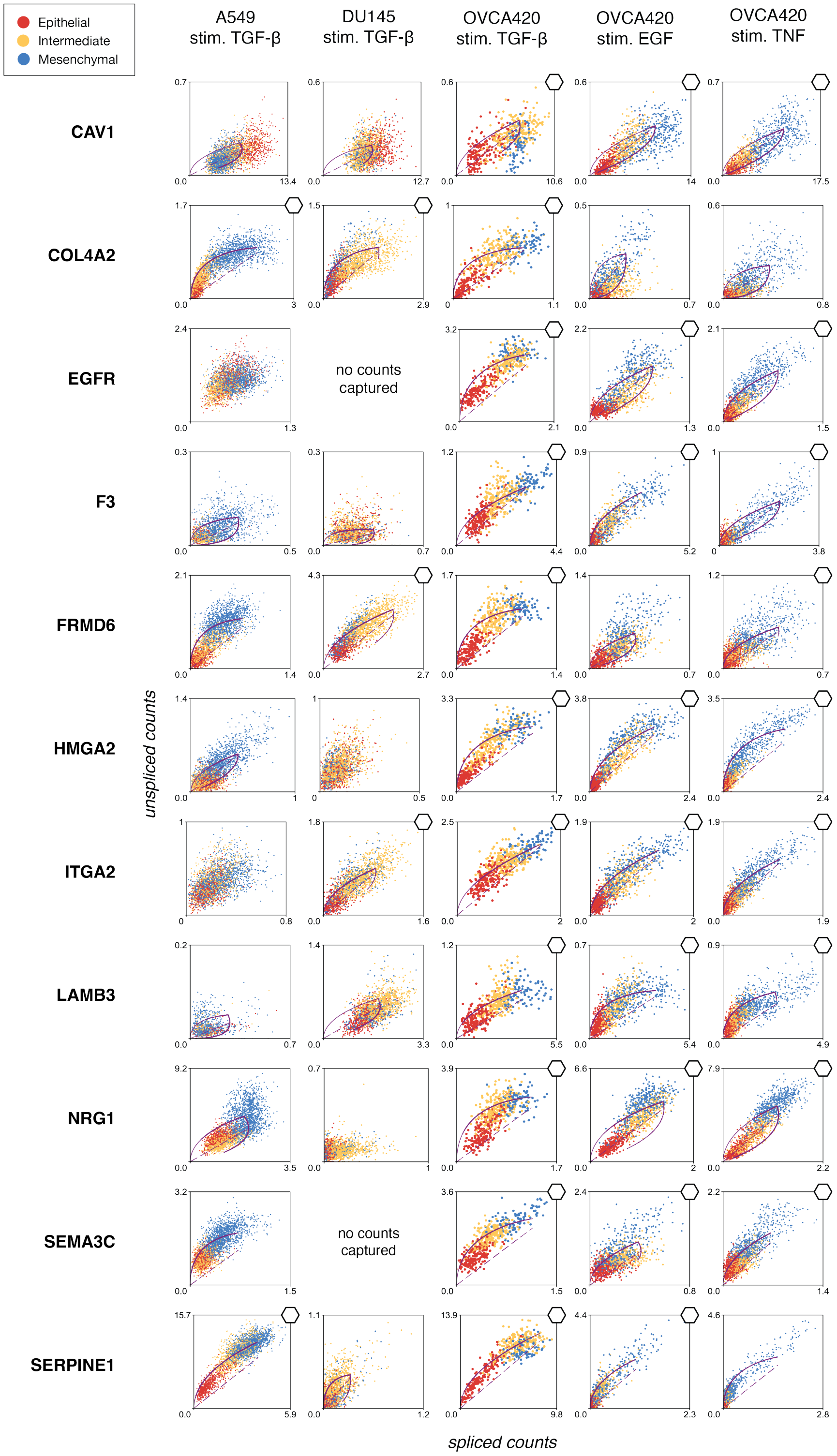

Supplementary Figure 8: ***Genes with upregulated RNA velocity in the majority of intermediate EMT states across samples from Cook and Vanderhyden, 2020.*** *Significant differential velocity for individual genes in specific samples is indicated by a rounded hexagon.*

a

$$\frac{dE}{dt} = -k_1 E I_1$$

$$\frac{dI_1}{dt} = k_1 E I_1 - k_2 I_1 I_2$$

$$\dots$$

$$\frac{dI_n}{dt} = k_n I_{n-1} I_n - k_{n+1} I_n M$$

$$\frac{dM}{dt} = k_{n+1} I_n M$$

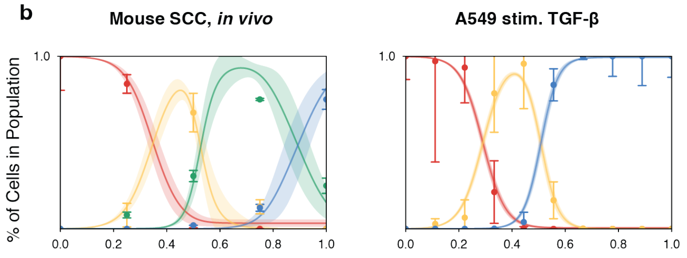

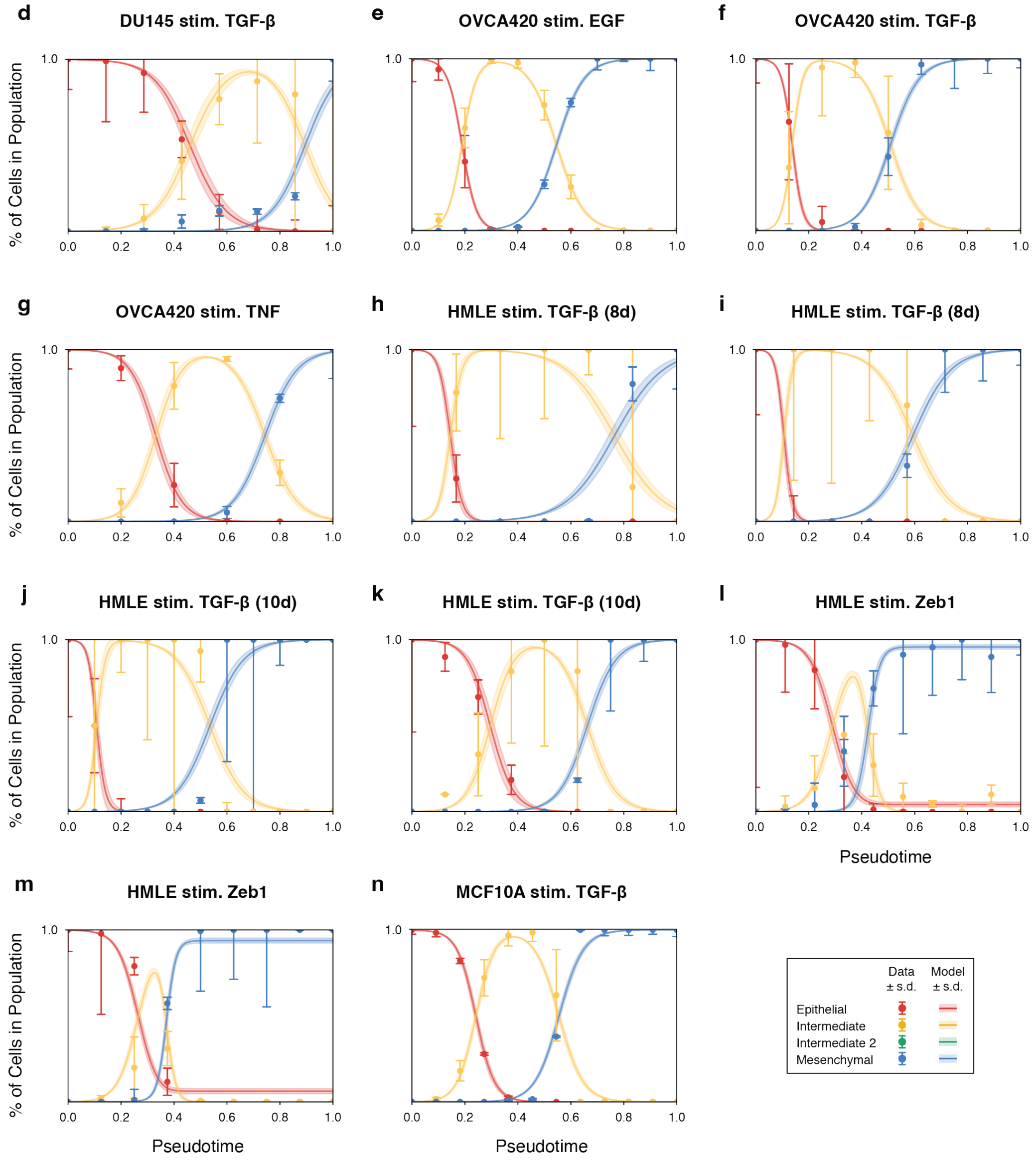

Supplementary Figure 9: ***Mathematical model fit to all scRNA-seq datasets. a.*** *Generalized mathematical model for n intermediate states.* ***b.*** *Model fits for each scRNA-seq sample following parameter inference, showing data vs. trajectory simulations with simulation parameters sampled from the posterior of each model.*

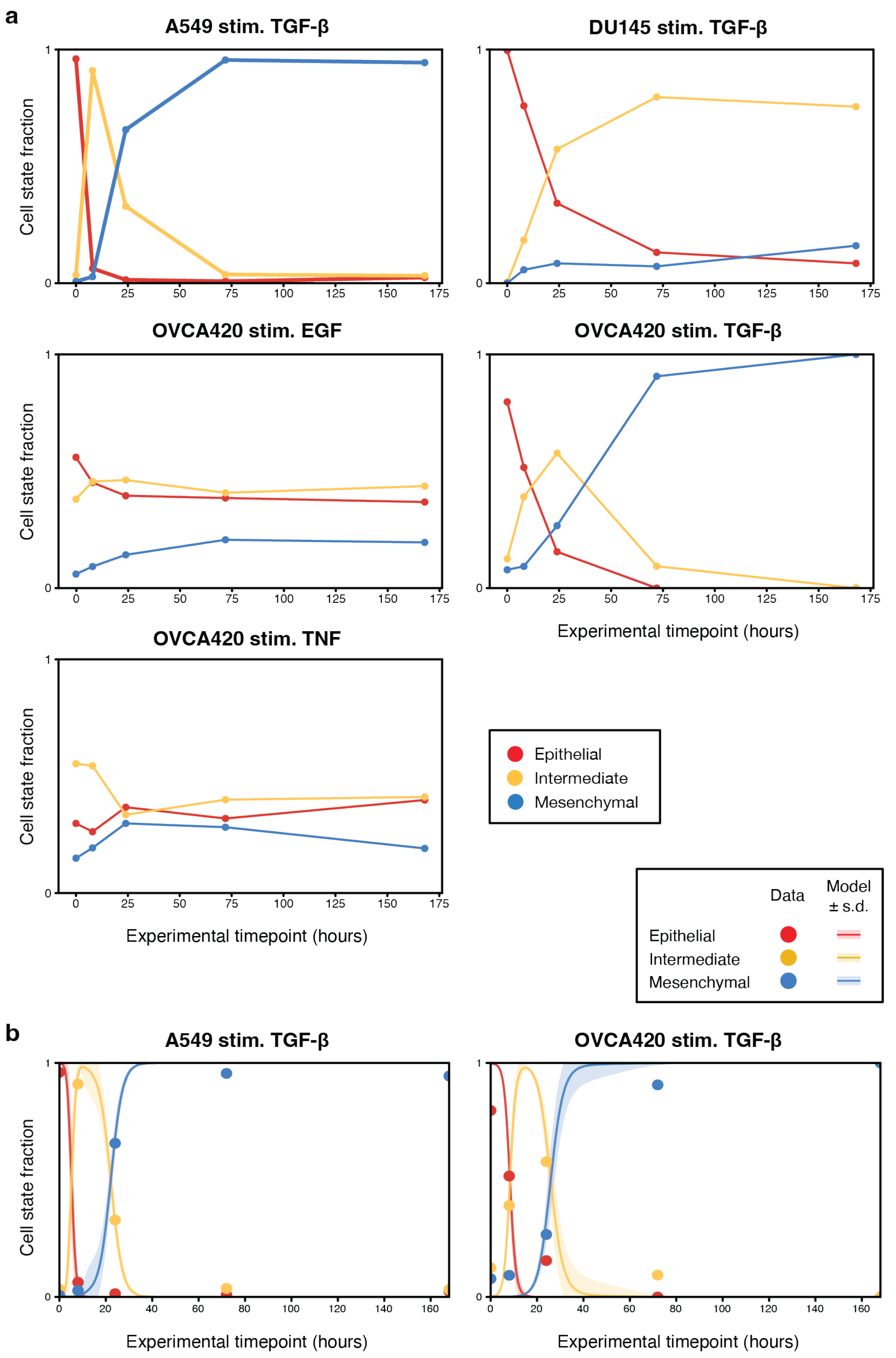

Supplementary Figure 10: ***Comparison of experimental time and pseudotime in scRNA-seq data. a.*** *EMT cluster proportions over real-time experimental data (available only for Cook and Vanderhyden, 2020 samples).* ***b.*** *Model fits across real-time, inferred from samples with high alignment between pseudotime and experimental time.*

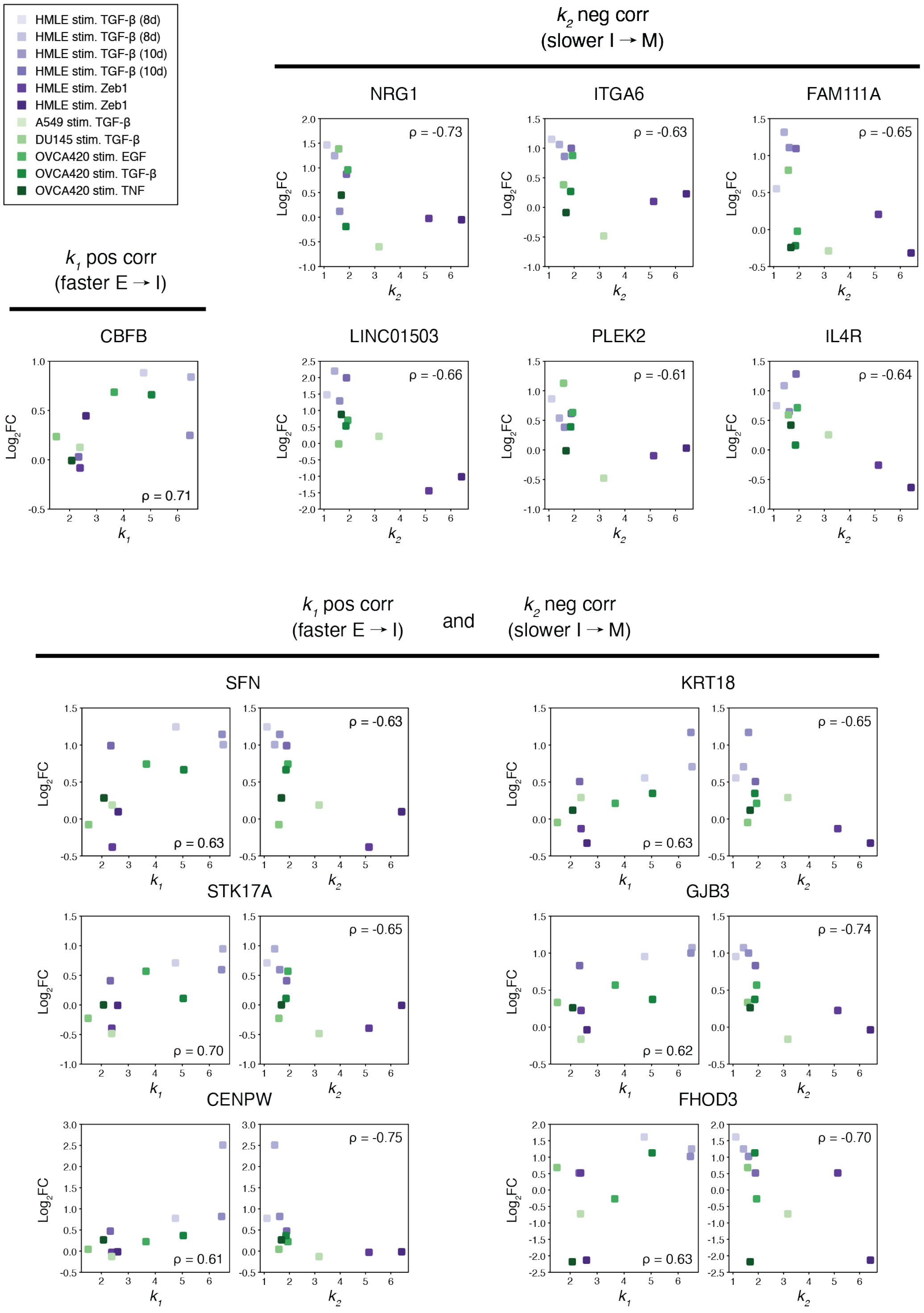

Supplementary Figure 11: ***Intermediate gene log$_2$FC correlated with fitted model parameters.*** *Significant Spearman's correlations ($p < 0.05$) were used to identify genes associated with stabilizing the intermediate state by increasing the transition rate into or decreasing the transition rate out of the intermediate state.*
